# Short- and Long-term Outcome After Interventional VSD Closure: A Single-Center Experience in Pediatric and Adult Patients

**DOI:** 10.1007/s00246-020-02456-2

**Published:** 2020-10-03

**Authors:** M. Bergmann, C. P. Germann, J. Nordmeyer, B. Peters, F. Berger, S. Schubert

**Affiliations:** 1 Department of Pediatric Cardiology and Congenital Heart Diseases, German Heart Center Berlin, Augustenburger Platz 1, 13353 Berlin, Germany; 2 Clinic for Pediatric Cardiology and Congenital Heart Defects, Herz- Und Diabeteszentrum NRW, Ruhr University of Bochum, Georgstraße 11, 32545 Bad Oeynhausen, Germany

**Keywords:** VSD, Congenital heart defect, Intervention, Cardiac catheterization

## Abstract

Interventional closure of congenital ventricular septal defects (VSD) is recording a continuous rise in acceptance. Complete atrioventricular block (cAVB) and residual shunting are major concerns during follow-up, but long-term data for both are still limited. We retrospectively evaluated the outcome of patients with interventional VSD closure and focused on long-term results (> 1 year follow-up). Transcatheter VSD closures were performed between 1993 and 2015, in 149 patients requiring 155 procedures (104 perimembranous, 29 muscular, 19 residual post-surgical VSDs, and 3 with multiple defects). The following devices were used: 65 × Amplatzer*™* Membranous VSD Occluder, 33 × Duct Occluder II, 27 × Muscular VSD Occluder, 3 × Duct Occluder I, 24 × PFM-Nit-Occlud®, and 3 × Rashkind-Occluder. The median age at time of implantation was 6.2 (0.01–66.1) years, median height 117 (49–188) cm, and median weight 20.9 (3.2–117) kg. Median follow-up time was 6.2 (1.1–21.3) years and closure rate was 86.2% at last follow-up. Complications resulting in device explantation include one case of cAVB with a Membranous VSD occluder 7 days after implantation and four cases due to residual shunt/malposition. Six (4%) deaths occurred during follow-up with only one procedural related death from a hybrid VSD closure. Overall, our reported results of interventional VSD closure show favorable outcomes with only one (0.7%) episode of cAVB. Interventional closure offers a good alternative to surgical closure and shows improved performance by using softer devices. However, prospective long-term data in the current era with different devices are still mandatory to assess the effectiveness and safety of this procedure.

## Introduction

Ventricular septal defect (VSD) is the most common congenital heart defect with a prevalence of 5.27 diseased children per 1000 live births [1]. Symptoms and therapy are dependent on the size of the defect and age of the patient. Possible complications of an untreated VSD can be pulmonary hyperperfusion and hypertension with Eisenmenger reaction after several years [2, 3]. In small children with large defects, early frequent treatment by surgical closure is preferred, whereas bigger children can be treated alternatively with transcatheter devices. However, both methods carry a potential risk of complete atrioventricular block (cAVB). Studies show cAVB occurs at a rate of 0.1–6.8% after interventional VSD closure [4–13] and < 2% after surgical VSD closure [14, 15]. Surgical closure can be considered for the majority of patients but disadvantages include the use of general anesthesia, sternotomy, and extracorporeal circulatory support, which results in longer recovery and hospital time [16–18]. Interventional closure can be done with sedation by a minimally invasive transcatheter application via a vein or artery and success is dependent mostly on the type of device. Therefore, the implications of the device used for VSD closure on outcome are presented in this study. Moreover, a special focus on the long-term outcomes of transcatheter VSD treatment, which are still limitedly reported in the international literature, shall be examined.

## Patients and Methods

We retrospectively reviewed 149 patients with congenital VSD who underwent transcatheter VSD closure between 04/1993 and 05/2015. Further inclusion criteria were perimembranous, muscular, multiple, and residual VSD after postoperative closure. We included both percutaneous and hybrid approaches and only unsuccessful implantations or VSD caused by myocardial infarction were excluded. Patient’s general data, device sizes, and echocardiography (ECHO) and electrocardiogram (ECG) examinations at the time of implantation were included. Primary endpoints were residual shunt and arrhythmia including cAVB, death, and explantation of device. Our study received ethical approval from the Charité–Universitätsmedizin Berlin (Ref-Nr. EA2/129/15).

### Catheterization Procedure and Follow-up

The catheterization procedure was performed following standardized operations protocols, as previously published [19]. After the intervention, all patients were assessed by ECHO and monitored by ECG as well as by a Holter monitoring device. A standard of care outpatient routine was conducted at time of discharge, roughly 1, 3, 6, and 12 months afterwards, and then yearly. Post-interventional treatment consisted of Aspirin (3–5 mg/kg/day per os) for 6 months. Follow-up data (> 1 year) were reviewed for residual shunting, arrhythmia, device dysfunction, re-intervention or explantation, and occurrence of death including cause of death.

### Statistical Analysis

Data were summarized using Microsoft Excel and presented as median (ranges), mean ± standard deviation, and percentage as appropriate. GraphPad Prism (Version 8) was utilized to evaluate data by unpaired *t* test, and *p* < 0.05 was considered to be statistically significant.

## Results

### Patient Data

A transcatheter closure of congenital VSD was performed in 149 patients with 155 procedures. The median age was 6.2 (0.01–66.1) years, median height 117 (49–188) cm, and median weight 20.9 (3.2–117) kg at time of intervention (see Table 1). The mean procedural time was 21.5 (4.4–120) min and the mean dose-area-product was 16035 (19-413500) milliGray per cm^2^. In our cohort, 143 patients had only one intervention, while 6 patients required multiple interventions (see Table 1). The location of the VSD was perimembranous (*n* = 104), muscular (*n* = 29), residual post-surgical VSDs (*n* = 19), and multiple defects (*n* = 3). A transfemoral approach was used in 142 patients and 7 hybrid interventions were included. The following devices were used: 65 × Amplatzer*™* Membranous VSD Occluder (VSD Memb), 27 × Amplatzer*™* Muscular VSD Occluder (VSD Musc), 24 × Nit-Occlud®, 3 × Amplatzer*™* Duct Occluder I (ADO I), and 33 × Amplatzer*™* Duct Occluder II (ADO II). The last two devices were used as “off-label.” Additional implantation data are summarized in Table 2.

**Table 1:** General patient data (*n* = 149)

Gender	
Male	80 (53.7%)
Female	69 (46.3%)
Approach	
Percutaneous	142 (95.3%)
Hybrid	7 (4.7%)
Amount of interventions	
Patients with 1 intervention	143 (96.0%)
Patients with 2 interventions	6 (4.0%)
Age at implantation*	6.3 (0.01–66.1) years
< 1 years	19 (12.8%)
1–10 years	70 (47.0%)
> 10–20 years	28 (18.8%)
> 20 years	32 (21.5%)
Height at implantation**	118 (49–188) cm
Bodyweight at implantation**	21 (3.2–117) kg

Data as median (range)

*For those patients with multiple interventions we used the date of the first intervention

**n = 146, due to missing data for 3 patients with Rashkind device

**Table 2:** Implantation data

	VSD Memb	ADO II	VSD Musc	Nit-Occlud	ADO I	Rashkind
	*n* = 65	*n* = 33	*n* = 27	*n* = 24	*n* = 3	*n* = 3
Age at implantation	11.4 (0.5–65.1)	2.2 (0–54.9)	1.9 (0.2–34.5)	9.4 (0.6–57.7)	9 (2.-46.8)	23.8 (12.1–26.9)
Height in cm	155 (65–188)	92 (49–183)	87 (56–176)	137.5 (66–185)	132 (87–184)	/
Bodyweight in kg	38 (6.7–117)	12.5 (3.2–104)	11 (4.1–64.5)	32.9 (5.9–87)	30 (12.1–72)	/
Device size in mm	6 (4–16)	5 (3–6)	8 (6–16)	8.5 (4–14)	8 (6–12)	15 (12–17) *

Data as median (range)

*n = 2, due to missing data

### Device Size

**Fig. 1:**
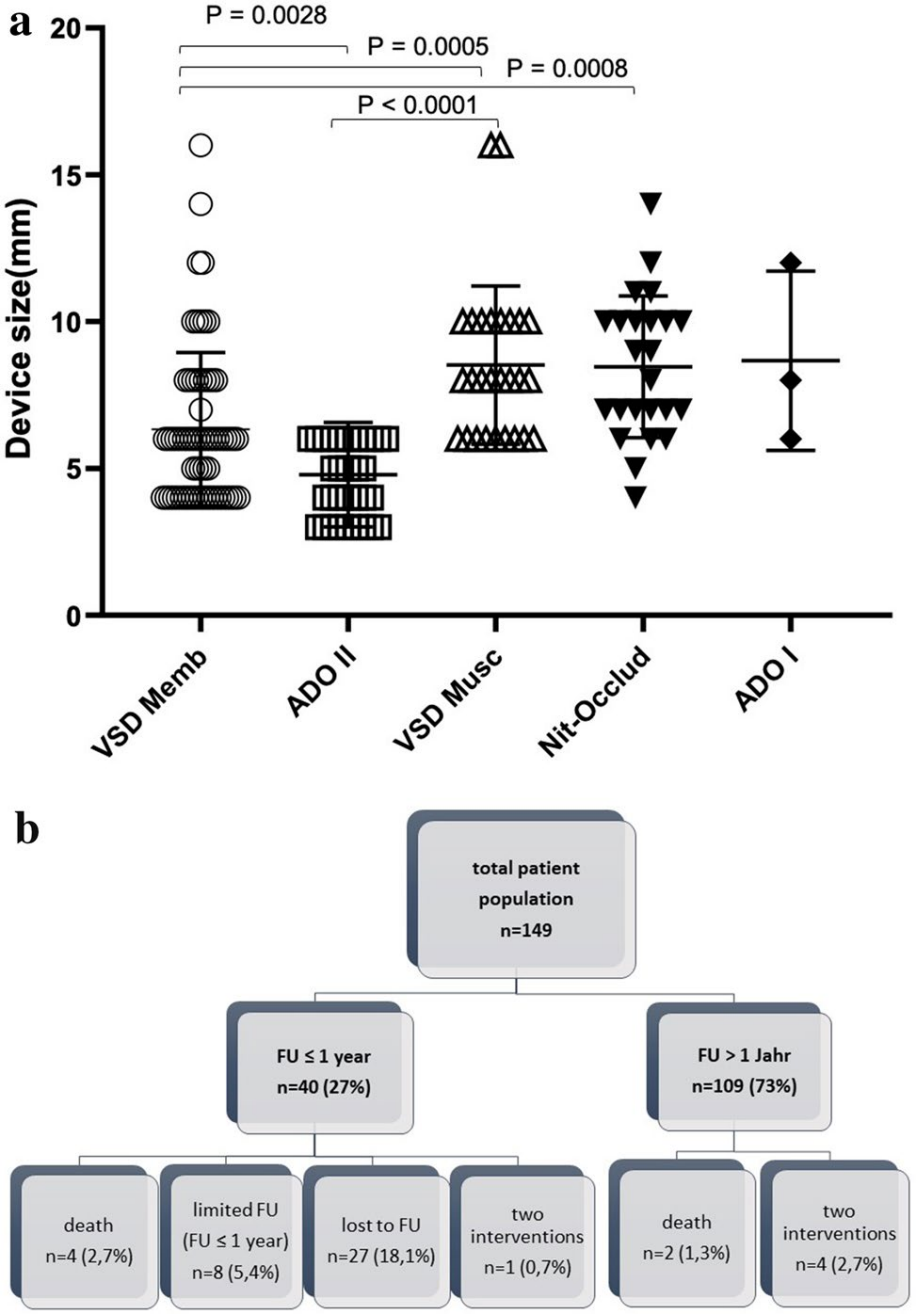
**a** Overview of the device size measured in mm for the different devices. Smaller devices had been used for Amplatzer***™*** VSD Memb and Duct occluder II. **b** Overview of the patient cohort and the supported follow-up (FU) time

Patients with a VSD Memb device were significantly older (median age of 11.4 years) at time of implantation, when compared to the other groups (VSD Memb vs. VSD Musc, *p* = 0.0028 and vs, ADO II, *p* = 0.01). ADO I and Rashkind-occluder were sidelined due to a small number of patients. The differences in age were congruent, leading to similar significant differences in height and bodyweight (VSD Memb vs. VSD Musc, *p* < 0.0001 and vs. ADO II, *p* = 0.003). In line with the differences in age, weight, and height, device size was also significantly different (see Fig. 1a).

### Follow-up

Overall results for the device groups as duration of follow-up, death, residual shunts, cAVB, and device explantation have been summarized in Table 3, and illustrated in Fig. 1b. Only four (2.5%) devices have been explanted (see also Table 3). We were able to collect follow-up data > 1 year in 109 out of 149 patients and achieved a median follow-up time of 6.2 (1.1–21.3) years with this cohort. The shortest follow-up in the ADO II group was due to the fact that these devices have been used for VSD closure since 2007.

**Table 3:** Results for duration of FU, residual shunts, cAVB and device explantation

	VSD Memb	ADO II	VSD Musc	Nit-Occlud	Rashkind	ADO I
	(*n* = 65)	(*n* = 33)	(*n* = 27)	(*n* = 24)	(*n* = 3)	(*n* = 3)
Lost to follow-up	*n* = 12 (18%)	*n* = 6 (18%)	*n* = 7 (26%)	*n* = 1 (4%)	*n* = 0	*n* = 1 (33%)
Follow-up > 1 year	*n* = 48 (74%)	*n* = 24 (73%)	*n* = 20 (74%)	*n* = 21 (88%)	*n* = 3 (100%)	*n* = 2 (67%)
Follow-up < 1 year	*n* = 5 (8%)	*n* = 3 (9%)	*n* = 0	*n* = 2 (8%)	*n* = 0	*n* = 0

	*n* = 53	*n* = 27	*n* = 20	*n* = 23	*n* = 3	*n* = 2
Follow-up (years) (median (range))	6.5 (0.5–13.1)	2.8 (0.1–6.3)	8.3 (1.8–13.4)	6.2 (0.1–12.8)	20.1 (15.6–21.3)	8.4 (5.8–11.1)
2nd device implantation^1^ 2nd device Residual shunts	1^#^ ADO II 5 (7.7%)	2^#^,* 2 × ADO II 5 (15.2%)	1^#^ Nit-Occlud 3 (11.1%)	1^#^ Nit-Occlud 7 (29.2%)	0	0
Death	2 (3.1%)	1 (3%)	2 (7.4%)	0	1 (33.3%)	0
cAVB	1	0	0	0	0	0
Device explantation	2	0	0	2	0	0

^1^Device implantation is part of total number of implantations

Reason for 2nd device: ^#^due to additional defect; *due to residual shunting

### Residual Shunt

**Fig. 2:**
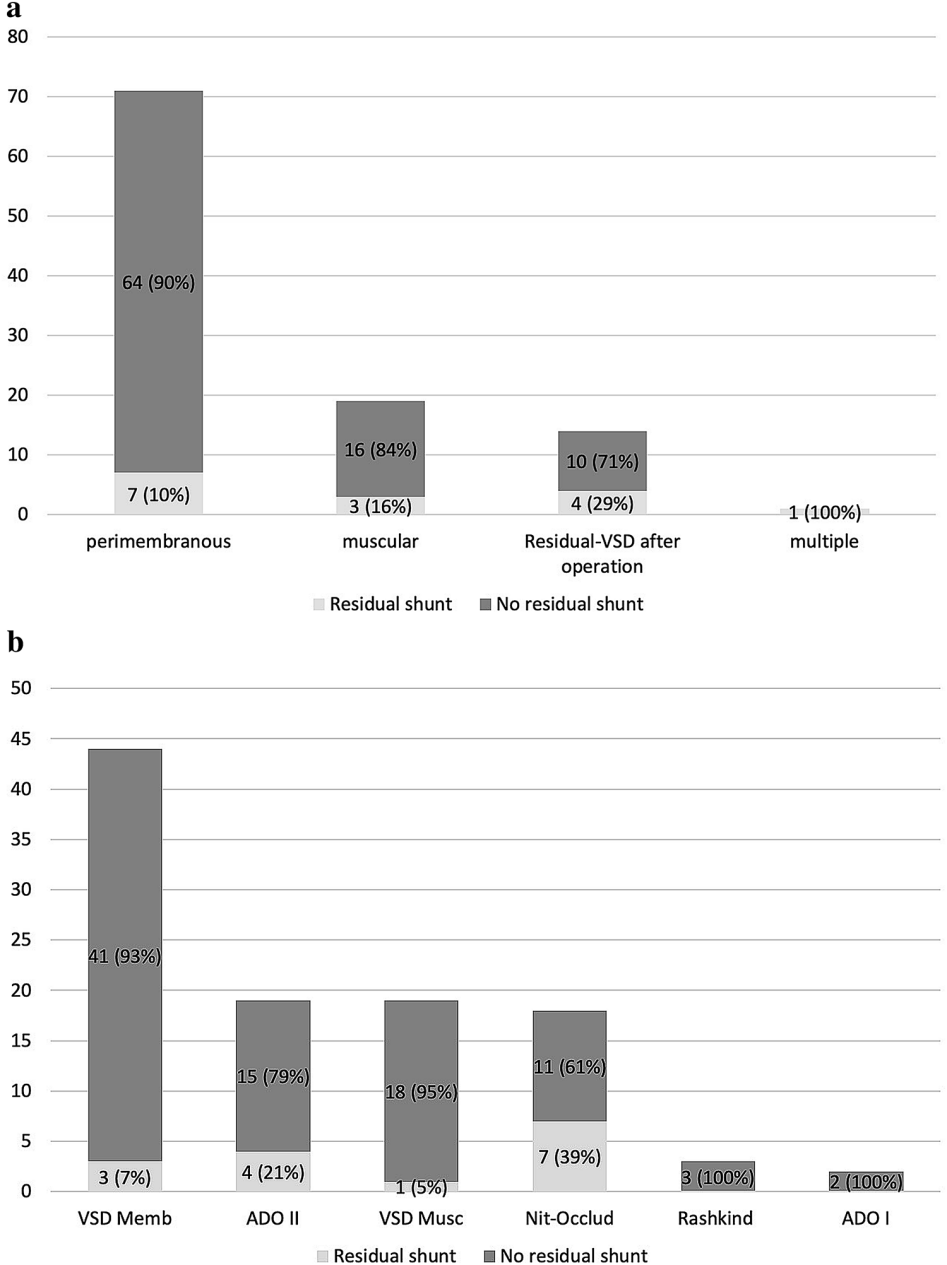
**a** Long-term results for residual shunt according to the defect location during long-term follow-up, number of patients (*n* in total = 109); **b** Long-term results for residual shunt, number of patients (*n* in total = 109) according to the implanted VSD device

The overall closure rate for all devices was in total 86.2 (61–95) % at best possible follow-up. The closure rate was influenced by the defect location and highest closure rates (> 90%) were achieved in closure of perimembranous VSD and lowest in residual/post-surgical VSDs (see Fig. 2a). Furthermore, perimembranous VSDs represented the largest group in our study. Sidelining the single multiple VSD, the lowest closure rate was achieved in residual VSDs after surgical treatment. Separating the closure rate according to the devices used and sidelining ADO I and Rashkind devices, the highest closure rates are seen in VSDs closed with VSD Musc (95%) and with VSD Memb (93%) (see Fig. 2b). Lowest closure rate of 61% was reported for Nit-Occlud®. One patient with a Nit-Occlud® developed a new small residual shunt 3 years after perimembranous VSD closure in the 40^th^ week of her pregnancy. This residual shunt showed no significant volume load over time and the device stayed in place.

### Arrhythmia During Short-term Follow-up

**Fig. 3:**
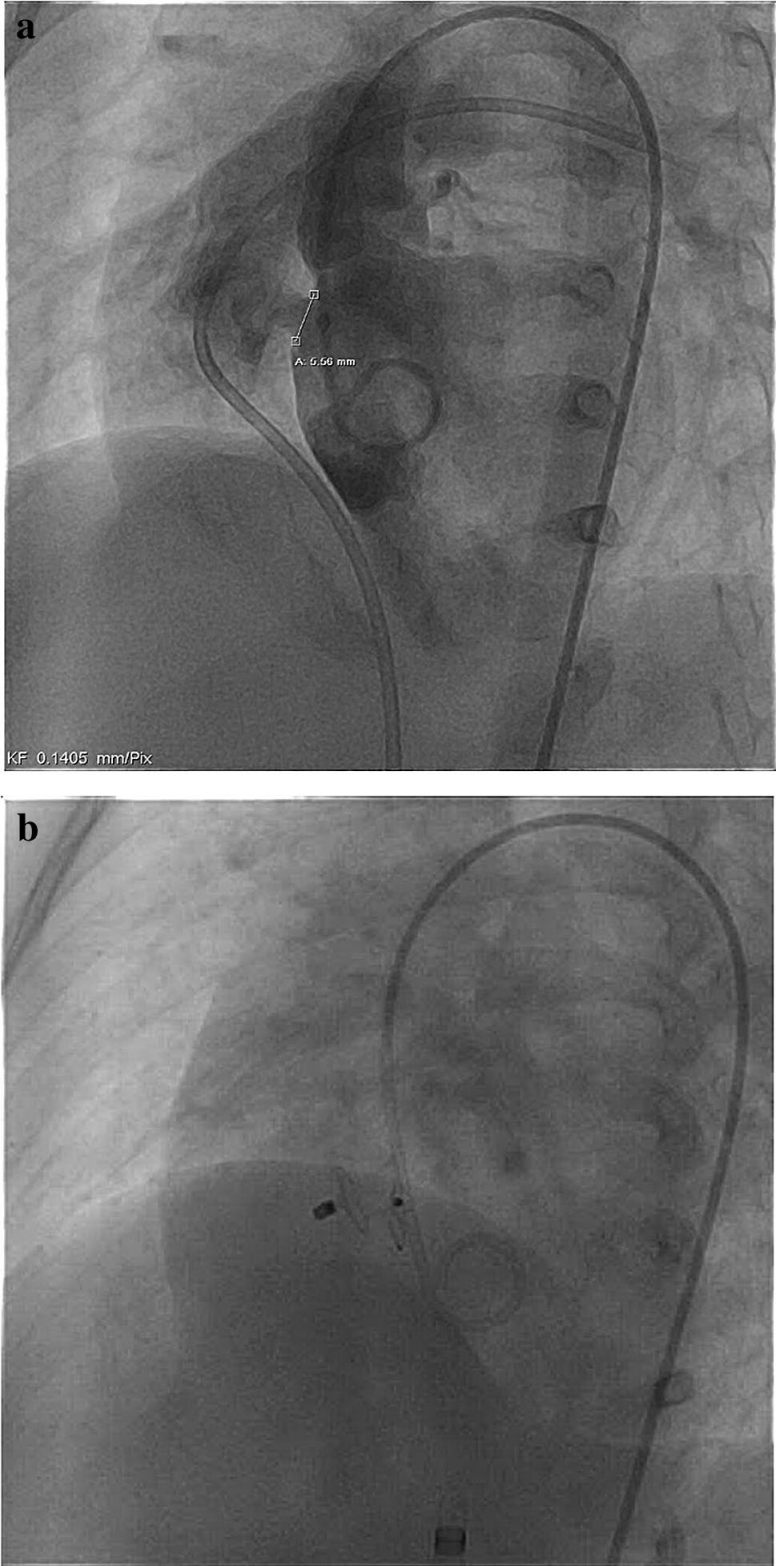
**a** Membranous VSD (diameter = 5.56 mm) with residual shunting; **b** Successfully implanted VSD Memb occluder (6 mm). “Oversized” configuration is visible in Fig. 2b, as indicated by an increased pressure related to the conduction tissue, which may be a cause for the early post-intervention detectable cAVB

**Fig. 4:**
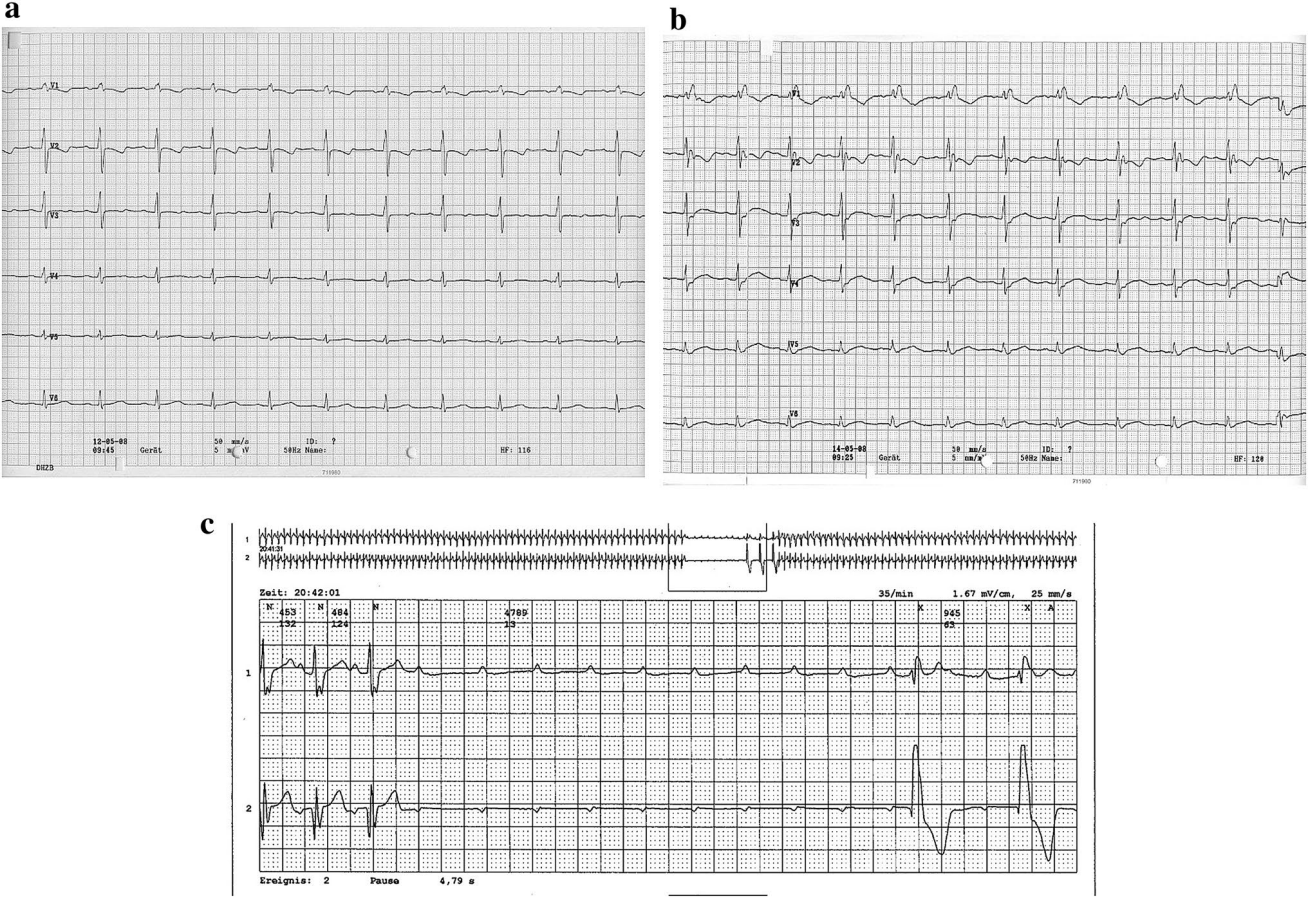
**a** ECG prior intervention; **b** ECG post intervention showing a complete RBBB; **c** Holter-ECG with cAVB at day 7 post intervention

As reported in Table 3, only 1/149 (rate of 0.7%) patient experienced a complete cAVB after percutaneous VSD implantation. In details, an 11-month-old girl with a membranous VSD (see Fig. 3a) was treated using a 6 mm VSD Memb device (see Fig. 3b). Residual shunting and aortic or tricuspid insufficiency were not seen, which was interpreted as an acceptable post-interventional result. Additionally, during and directly after the intervention, there was only a new right bundle branch block (RBBB) detectable but without events of cAVB (see Fig. 4a, b), and the child was discharged 48 h after the intervention. An acute cAVB occurred 7 days after VSD closure in a Holter examination during follow-up (see Fig. 4c). As a consequence, the girl was sent to surgery for device explantation and surgical VSD closure was performed with a pericardial patch. The postoperative persisting cAVB was treated by temporary DDD-Pacing and triple administration of Prednisolon, with full recovery within 5 days and in the follow-up. Remarkable are the signs of oversizing (see Fig. 3b), which may be responsible as risk factor for cAVB, especially with the use of the VSD Memb device and prelude for a cAVB might be a new RBBB during or immediately after placement of the device.

### Arrhythmia During Long-term Follow-up

All patients were constantly examined for cardiac arrhythmias during follow-up with the use of ECG and Holter-ECG. Arrhythmia occurred in 4/109 patients (rate of 3.7%) during long-term follow-up (> 1 year) with need for treatment (medication, ablation, or pacemaker implantation). Bradyarrhythmia was detected in only one patient. This patient had an ADO I device and a RBBB, extrasystoles, and bradycardia occurred during long-term FU. This was stable for the last 7 years and RBBB was mainly caused by an underlying diagnosis of Tetralogy of Fallot, peripheral vascular disease, and status post correction. This was not related to the VSD itself because it was already detected prior to the implantation. Two other adult patients with a VSD Musc and a Rashkind device experienced tachyarrhythmia (atrial and ventricular extrasystolia and atrial fibrillation/flutter). In one patient, an underlying severe restrictive cardiomyopathy and supraventricular extrasystoles were detected before VSD closure and this patient needed an ablation and cardiac resynchronization therapy (CRT)-pacemaker 3 months after VSD closure in order to improve anti-tachycardiac treatment. Another patient suffered atrial fibrillation after implantation of the ADO II device. No explantation of a device due to arrhythmia and cAVB occurred during the long-term follow-up period (median 6.2 years).

### Mortality

Mortality occurred in 6/149 (rate of 4%) patients during follow-up, of which none were related to the device. A procedure-related mortality has been reported in only one patient involving a 4-day-old girl who died after a hybrid approach for residual VSD closure. Perforation of the left ventricular heart wall occurred during the implantation of a 4 mm VSD Musc device after complex corrective surgery of aortic arch, which was resolved during cardiopulmonary bypass (CPB). However, the patient needed extracorporeal membrane oxygenation (ECMO) due to low cardiac output syndrome afterwards and later weaning from ECMO was not possible. Bleeding complications caused major morbidity and ECMO support was stopped after 24 days.

All other mortalities (*n* = 5) were neither device- nor procedure-related deaths. One female patient died 41 years after surgical VSD closure and 20 years after interventional VSD closure with a Rashkind device due to tachyarrhythmia (VT) without device dysfunction. Another adult patient died at the age of 41 years, 2.1 years after VSD closure with a VSD Memb device due to severe heart failure (HFrEF). Multi-organ failure including chronic kidney failure was the cause of death for another male patient (77 years old) with the implanted VSD Memb device in situ. Another 7-month-old child with a borderline biventricular anatomy achieved a cardiac decompensation 2 weeks after corrective surgery including hybrid VSD closure with a VSD Musc device. Conversion to a functionally univentricular heart as rescue procedure was not sufficient. A patient with transposition of the great arteries (TGA) was treated to close a residual VSD using a VSD Musc device and died 4 months later at the age of 2 years. It was impossible to find the cause of death in her medical records, but best possible follow-up revealed no signs of arrhythmia nor device malfunction.

## Discussion

Today, percutaneous transcatheter VSD closure is an established treatment alternative. However, the success rate depends on the relation of VSD diameter and height/weight of the child, as well as on the distance to the aortic and tricuspid valve [20]. Although relevant residual shunting and bradyarrhythmia (especially cAVB) are major concerns, only limited data are available with long-term follow-up for these issues [10, 19, 21–23]. In order to demonstrate the safety and performance of VSD devices in the long run, we focused on patients with at least 1-year follow-up after the procedure, since many publications only include short- or middle-term follow-up data [8, 23–25]. According to these data, we could achieve a long-term follow-up period of 6.2 (1.1–21.3) years. We included 155 procedures in 149 patients with 95.3% (142/149) by percutaneous and 4.7% (7/149) by hybrid approach. 47% (70/149) of the procedures were performed in the age between 1 and 10 years. Six different devices could be compared. This is in contrast to the majority of publications, analyzing only one type of device [6, 7, 19] and only during short- or mid-term follow-up [4, 5].

### Residual Shunt

A residual shunt rate of 3 to 29% has been reported in the past decades [4, 13, 22, 26, 27].

The overall residual shunt rate of 13.8% in our long-term cohort seems to be moderate and tolerable, but we have to emphasize that residual shunts were small and did caused relevant volume load over time. Nevertheless, the group with the Amplatzer*™* Membranous and Musc VSD occluder showed the highest closure rate of 93–95%, perhaps due to a better correlation between the diameter of the VSD and height of the child. Base on the technical issues of sheath size, possibility for implantation success, and complications, ADO II devices have been especially used in smaller children [19]. Moreover, the VSD occluder required a longer follow-up time in contrast to the more flexible and “off-label” used ADO II, thus, probable reasons for the higher closure rates observed for these devices [5, 6, 8, 13, 23, 26, 28–30].

The residual shunt rate of 39% for the Nit-Occlud® group observed in our cohort was higher in comparison to other studies, e.g., Haas et al. (5% at 6 month, 3% at 12 month, and no residual shunt after 4 years), Nguyen (5.9–8.7% after 6 months), El Shedoudy et al. (2.5% after 1 year), and Odemis et al. (15% after 12.3 months). Remarkably, a new small but detectable shunt was again detected in one of our patients with the Nit-Occlud® 3 years after the procedure during pregnancy. This reopening of the shunt might be a result of increased cardiac output and ventricular dilatation during pregnancy [7, 9, 31, 32]. Therefore, residual shunting appears to be related to the device type and location of the VSD. We could demonstrate that post-surgical VSD and the Nit-Occlud® device lead to higher residual shunt rates. Comparing different devices at different follow-up periods may be a reason for higher residual shunt rates. This has also been reported by Carminati et al., who observed a residual shunt rate of 17% at a median follow-up time of 2 (0.5–10) years in 430 patients with 6 different devices [4].

### Arrhythmia

The most feared complication of transcatheter VSD closure is cAVB with a reported incidence of 0–6.4% [7, 25, 28, 30, 32] and cAVB after surgical VSD closure has been reported with a prevalence of 1–5% [16, 33–35]. More current studies have reported cAVB rates between 1.25 and 1.4% for transcatheter VSD closure as a comparable risk after operation [9, 31, 36–39]. According to our data, we observed only 1/149 (rate 0.7%) patient with cAVB for all different VSD devices investigated during > 6 years of follow-up, which is a lower rate than published data to date for interventional and surgical approach. Cinteza et al. report early postoperative cAVB in contrast to an unclear or later manifestation of a block after the intervention. According to their understanding the early events are also device related and may be due to the thickness of nitinol wires [40]. The majority of cAVB are detected 2–7 days after the procedure, but late cAVB after 2–4 weeks or 10–20 months have also been reported in the literature [41–48]. In addition, a systematic review by Yang et al. reported 107 out of 4394 patients (2.4%, 95% CI of 1.6–3.2) needed a permanent pacemaker after interventional procedure with a higher rate in younger children. In 86% (92/107) of these cases, the block manifested in the first week [27]. Although other studies also described late appearances of a cAVB [4, 49], long-term follow-up data are rare. Moreover, Carminati et al. summarized from their data that regular ECG controls are essential during follow-up [4, 50].

It appears to be possible that mechanical trauma, compression, inflammation, edema, and consecutive scarring resulting in a cAVB can be reduced, especially with the more flexible and softer devices such as ADO II [51–53]. Additionally, the use of oversized Amplatzer*™* VSD devices needs to be avoided. We retrospectively speculate that this was a cofounding factor in our one patient who developed early cAVB. Other arrhythmias, including premature heart beats, tachycardia, and atrial or ventricular fibrillation are known risk factors for relevant temporary arrhythmia of 10.6% (95% CI of 8.4–12.7) and persistent arrhythmia of 3.1% (95% CI of 2.0–4.1) [27]. In our follow-up cohort, we observed 2.9% (3/105) tachyarrhythmia with an indication for therapy as tachycardia or atrial fibrillation. However, we did not observed a correlation to the procedure or device itself.

### Mortality

We also analyzed all cases of mortality in both the short- and long-term follow-ups. Procedure-related mortality-rate was a low 0.7% (1/149 patients), and this particular patient died due to a complication from a hybrid approach for complex VSD closure. There was no device- or procedure-related mortality for the transfemoral approach. Two other children died within the first year of intervention due to treatment for univentricular or complex congenital cardiac disease, which was again not procedure- or device-related. In the long-term follow-up, another 3 patients died due to progression of biventricular heart failure or multiple organ failure with acute decompensation. In the literature, procedure-related deaths are rare (between 0 and 3%) [28, 34]. Wang et al. observed that the risk for major complications was significantly higher in children younger than 3 years old. Therefore, they recommend operations for patients with a VSD size larger than 10 mm or prolapse of the aortic valve [10]. In general, the literature shows different thresholds for percutaneous VSD occluder according to the increased risk of complications for patients below the threshold of 10 kg [8, 13, 54, 55]. Finally, all of these authors concluded that percutaneous transcatheter VSD closure is safe and effective. This is confirmed by our analysis pointing out no (0%) device- or procedure-related mortality for the transfemoral approach.

Meta-analysis by Saurav et al. comparing operative and interventional VSD closure showed comparable effectivity and safety [17]. Today, radiation dosage during transcatheter VSD closure appears not to be a relevant risk factor anymore [4, 7]. Moreover, hospital stay after catheter procedures is about 2.2 days shorter, costs less, and the general risks and complications associated with operations involving the heart lung machine and sternotomy can be avoided [16–19, 56–60]. Finally, it should not be left unmentioned that in general, the operative VSD cohort includes children who are younger with earlier symptoms and larger VSDs [27].

## Limitation

A limitation of our study is the retrospective and single-center design. As successful implantation was an inclusion criteria for our study design, we did not analyze the success rate for this interventional procedure. Future prospective multicenter studies should reinforce the results with more powerful data. Finally, the long follow-up time of more than 20 years causes difficulties in comparability between the patients in our cohort, due to the development of new devices over this period. This means that different devices were not available for all patients at all times investigated. On the other hand, the long period of over 2 decades increases the validity of the results showing effectiveness and safety.

## Conclusion

In conclusion, interventional VSD closure is becoming more important with growing experience and improving devices, and appears to be safe and effective for the majority of patients. Our results show that percutaneous VSD closure is a well-established procedure with a low severe adverse event rate. In our long-term follow-up group of 109 patients, we can report absence of cAVB and no late deaths were related to the procedure or the device. All residual shunts were small and did not caused relevant volume load or ventricular enlargement. Moreover, we identified the following two risk factors for residual shunting: VSDs after surgical closure and use of the Nit-Occlud® device. The only procedure-related death occurred in a patient treated by hybrid intervention. Based on common assumptions in the international literature, we speculate that the use of an oversized Membranous Amplatzer*™* occluder may be the major reason for the development of cAVB in our patient. However, a prospective or randomized comparison of the impact from the device itself on the efficacy or complications is still needed.

To date, transcatheter VSD closure is being established as a safe and effective alternative for selected patients. The ongoing optimization of the existing devices, as well as development of newer and more flexible devices, such as the Lifetech Konar-MF*™* VSD Occluder, may have the potential to further reduce the risk [61, 62].
